# Comparative Immunogenicity of 7 and 13-Valent Pneumococcal Conjugate Vaccines and the Development of Functional Antibodies to Cross-Reactive Serotypes

**DOI:** 10.1371/journal.pone.0074906

**Published:** 2013-09-23

**Authors:** Lindsay R. Grant, Sarah E. O’Brien, Polly Burbidge, Mitch Haston, Marta Zancolli, Lucy Cowell, Marina Johnson, Robert C. Weatherholtz, Raymond Reid, Mathuram Santosham, Katherine L. O’Brien, David Goldblatt

**Affiliations:** 1 Center for American Indian Health, Johns Hopkins Bloomberg School of Public Health, Baltimore, Maryland, United States of America; 2 Institute of Child Health, University College London, London, United Kingdom; Health Protection Agency, United Kingdom

## Abstract

**Background:**

Protection against disease or colonization from serotypes related to those in pneumococcal conjugate vaccines (i.e. cross-protection) vary by serotype; the basis for this variation is not understood. The 13-valent pneumococcal conjugate vaccine (PCV13) replaced 7-valent conjugate (PCV7) in the USA in 2010 allowing assessment of PCV7 and PCV13 immunogenicity and functional cross-protection in vitro.

**Methods:**

Post-primary, pre-booster and post-booster sera from American Indian children receiving exclusively PCV7 or PCV13 were collected. IgG was measured by ELISA for 13 vaccine serotypes; functional antibody was assessed by opsonophagocytic killing assays for serotypes 6A/B/C and 19A/F.

**Results:**

Post-primary IgG geometric mean concentrations (GMC) for serotypes 4 and 9V were lower in PCV13 recipients while 19F GMCs were higher. Only 19F differences persisted after receipt of the booster dose. Functional antibody activity was higher among PCV13 recipients for 6A, 6C, 19A and 19F (p<0.04), and among PCV7 recipients for 6B (p = 0.01). Following PCV7, functional antibodies to 6A but not 19A were observed. High levels of 6C functional activity were seen after PCV13 but not PCV7.

**Conclusions:**

Functional antibody activity against 6A/B/C and 19A/F suggest that PCV13 is likely to control the 19A disease and 6C disease remaining despite widespread use of PCV7.

## Background

The seven-valent pneumococcal conjugate vaccine (PCV7, Prevnar, Pfizer) was licensed and introduced in 2000 into the United States (US) infant immunization schedule; doses are administered at 2, 4, 6 and 12–15 months of age. The incidence of invasive pneumococcal disease (IPD) from the vaccine serotypes (4, 6B, 9V, 14, 18C, 19F and 23F) fell rapidly following PCV7 introduction, although serotype-specific efficacy varied by serotype (87% (19F) to 100% (9V)) [1]. For non-invasive disease, serotype specific efficacies have shown even greater variation; in a PCV7 efficacy trial of acute otitis media (AOM) efficacy ranged from only 37% for serotype 19F to 79% for serotype 6B [2]. This variability in efficacy was not associated with significant differences in absolute antibody concentrations but was linked to increased antibody requirement for killing of 19F, possibly explained by the thickness of the 19F capsule and increased resistance to C3 deposition [3].

In addition to serotype specific efficacy, there has been interest in cross-protective immunity induced by the constituents of PCV7 to closely related serotypes. Prior to the widespread use of PCV7, it was unclear whether 6B and 19F induced antibodies would impact 6A and 19A carriage and disease respectively. Following implementation, here too variation has been seen. Use of PCV7 has resulted in 76% effectiveness against 6A IPD, with no impact on 6C disease [4], and an estimated 26% effectiveness against IPD caused by 19A [1]. So, unlike the ability of 6B-induced antibodies to cross-protect against 6A disease, antibodies inducted by 19F antigen present in PCV7 have provided limited cross-reactive protection against 19A disease [5]. To go beyond the disease protection capacity of PCV7, extended valency vaccines have been developed which include some or all of six additional capsular polysaccharides (1, 3, 5, 6A, 7F and 19A). One of these vaccines, designated PCV13 (Prevnar-13, Pfizer), is licensed in the US and replaced PCV7 in the first quarter of 2010 for routine use; it and the 10-valent product (PCV10, GlaxoSmithKline) are used in many countries around the world. The US implementation of PCV13 provides the opportunity to more deeply investigate the cross-reactive potential of the serogroup 6 and 19 antigens included in the vaccine and thus anticipate the likely impact this vaccine will have on disease, especially in populations with a high disease burden.

American Indians living on or around the Navajo and White Mountain Apache reservations in the Southwest US have increased rates of pneumococcal nasopharyngeal (NP) colonization and disease compared to the general US population [6], [7]. However, PCV7 has had a profound impact on reducing IPD due to vaccine serotypes to negligible levels [8], [9]. The presence of cross protection to 6A by the 6B conjugate that is contained in PCV7 has been apparent. During the PCV7 routine use era, population-based active surveillance among Navajo and White Mountain Apache communities demonstrated that serotype 6A IPD rates decreased compared to pre-PCV7 use era (2006–2008 vs. 1998–2000) although NP colonization and IPD rates caused by serotype 6C increased [4]. Invasive disease from 19A has also increased over time among US children (including the Navajo and White Mountain Apache) and emerged as the leading serotype causing IPD in other US populations [8], [10]. As expected based on trends in 19A IPD, 19A also became a frequent cause of NP colonization; from 2006–2008, 19A was among the five most frequent serotypes isolated from Navajo and White Mountain Apaches enrolled into a longitudinal, household-based study of pneumococcal colonization [11]. Interestingly, vaccine-type 19F, unlike other PCV7 serotypes, continues to circulate as a colonizing serotype and is occasionally detected as a cause of IPD among the Navajo and White Mountain Apaches.

In March and April 2010, PCV13 replaced PCV7 among Navajo and White Mountain Apache infants thereby providing an opportunity to evaluate the comparative immunogenicity of PCV7 and PCV13. Of particular interest was the ability of sera taken following administration of PCV7 or PCV13 to kill serotypes 6B and 19F and related serotypes 6A, 6C and 19A as this would provide information on the likely degree of direct and cross-protection afforded by PCV13 in this high risk population.

## Methods

### IRB Approval & Consent

The Institutional Review Boards of the Johns Hopkins Bloomberg School of Public Health and the Navajo Nation approved this study. Tribal approval was obtained from the Navajo Nation. Parents and guardians signed a written informed consent document for each child participant.

### Study Design

An observational immunogenicity study of PCV7 and PCV13 was nested within a community-based study of pneumococcal NP colonization conducted from January 2010 to March 2012 enrolling American Indian children and their families who lived on or near Navajo Nation. The population of Navajo Nation is more than 250,000 people who live on 27,000 square miles of land that is divided into nine service units as defined by the Indian Health Service (IHS). From the service units of Chinle, Fort Defiance, Gallup and Shiprock, families and their infants 7–24 months of age were recruited at the primary IHS health facility on each service unit for enrolment into the colonization study. Infants who received three doses of PCV7 or three doses of PCV13 as part of the routine immunization program were eligible for the nested immunogenicity study, which was determined by review of the infant’s medical record. Following consent by the parent or guardian, blood was collected after primary immunization, prior to and/or following the booster dose. The goal was to collect 600 bloods total: 100 bloods at each of the three time points from the same or assorted patients who received either PCV7 (300 specimens) or PCV13 (300 specimens). Therefore, children could contribute to any or all of the sampling time points depending on their immunization status.

The ideal blood collection criteria are: (1) at least 28 days and no more than 112 days had to separate the primary series doses and (2) the booster dose had to occur at least 120 days after completion of the primary series. Post-primary and post-booster blood draws had to be completed within 98 days after the last dose of the primary series and within the first year of life or after the booster dose and before 20 months of age. Pre-booster blood was drawn anytime in the 14-day window prior to the booster dose. There were no additional exclusion criteria for a specimen to be in the analysis.

### Laboratory Procedures

Approximately 4 mL of blood collected by venipuncture, separated and serum stored at −80°C. Serum was assayed for antibodies to 13 vaccine-type capsular polysaccharides (1, 3, 4, 5, 6A, 6B, 7F, 9V, 14, 18C, 19A, 19F and 23F) at the University College London, Institute of Child Health, a World Health Organization (WHO) reference laboratory for pneumococcal serology. Sera were assayed using the WHO reference enzyme-linked immunosorbent assay (ELISA) following adsorption with cell wall polysaccharide and 22F polysaccharide at a concentration of 10 *µ*g/mL as previously described [12], and in detail at http://www.vaccine.uab.edu/ELISA%20Protocol.pdf. To evaluate functional antibody to serotypes 6A, 6B, 6C, 19A and 19F, a subset of post-primary sera from PCV7 and PCV13 recipients were selected, matching for anti-19F or anti-6B concentrations. Sera were analyzed by a multiplexed opsonophagocytic assay (OPA, titer ≥1∶8 considered positive) [13]. To assess the contribution of type specific antibody to killing of cross-reactive antigens, sera were retested by OPA after adsorbing sera with purified 19F, 19A, 6A, 6B or 6C capsular polysaccharide (ATCC for 19F and 19A and Statens Serum Institute for 6A, 6B, and 6C) one at a time and reported as the titer where 50% of bacteria were killed.

### Statistical Analyses

Serotype-specific ELISA geometric mean concentrations (GMCs) were calculated for each blood draw and comparisons made between those who received PCV7 or PCV13. Serotype-specific IgG values were log-transformed and used in Generalized Linear Models with log(IgG) values following a Gaussian distribution to adjust for differences by vaccine group in the number of days between the last dose that occurred immediately prior to the blood draw. A robust variance estimator was used in the model to account for dependent observations when a child contributed more than one specimen across collection times. The adjusted IgG values from the models were then used to calculate the GMC and corresponding 95% confidence intervals. The coefficient in the model for vaccine group represented the difference in the adjusted GMCs between the vaccine groups. The model tested inclusion of an interaction term between vaccine group and time between dose and the blood draw to account for differences in antibody concentration between vaccine groups. The immune response to the booster dose was measured by comparing the pre- and post-booster GMCs for each serotype by vaccine. A p-value <0.05 was considered statistically significant. We calculated the proportion of participants who achieved the threshold antibody concentration used in evaluation of vaccines against type-specific IPD; we used 0.35 *µ*g/mL (the value from the combined results of the American Indian, Northern California and South African PCV7 efficacy trials [14]) and 1.0 *µ*g/mL (the value derived from the American Indian trial alone [15]).

Serum samples from PCV7 or PCV13 recipients were matched on 6B and 19F ELISA concentrations, resulting in a subset of post-primary sera that were tested by OPA for functional antibody to serotypes 6A, 6B, 6C, 19A and 19F. Sera were matched using five IgG concentration (*µ*g/mL) categories of low (<0.5), medium-low (0.5–0.99), medium (1.00–4.00), medium-high (4.01–10) and high (>10.00). Geometric mean titers (GMTs) were calculated and compared. Correlation between the OPA GMTs and ELISA GMCs was evaluated by a Pearson correlation coefficient (r). The percentage of functional antibody adsorbed by free capsular polysaccharide was compared between the PCV7 and PCV13 vaccine recipients using Fisher’s exact test. GMTs were compared between vaccine groups on the log scale for individual serotypes by a paired two-sample t-test for data following a normal distribution. The non-parametric Wilcoxon signed-rank test was used to compare GMTs that were not normally distributed on the log-scale. Analyses were completed using STATA (version 11; College Station, TX). Data are available from the authors upon request.

## Results

In total 561 sera were collected (159 post-primary, 222 pre-booster, 180 post-booster) between April 2010 and October 2011. Of the 561 collected sera, 209 children contributed one specimen, 73 children provided two specimens (N = 146) and 17 children gave three specimens (N = 51). Table 1 provides comparisons of sera obtained from PCV7 and PCV13 recipients that contributed to the immunogenicity analysis. Sera that were not included in analyses failed to meet criteria related to intervals of time between vaccine doses, time between dose and blood draw, number of vaccine doses before the blood draw, mixed use of PCV7 and PCV13 and the age at blood draw (Figure S1). There were 117 post-primary, 157 pre-booster and 133 post-booster (total = 407) sera that met the inclusion criteria for analysis. Children who received three PCV13 primary doses were younger at each dose than PCV7 recipients (p<0.01), PCV13 children had shorter intervals of time separating some doses (p<0.008) and fewer days between the previous dose and the blood draw compared to PCV7 children (p<0.009; Table 1).

**Table 1:** Dosing and draw time characteristics of study participants contributing to immunogenicity analysis.

	Post-Primary Series			Pre-Booster Dose			Post-Booster Dose		
Characteristics	PCV7(n = 32)	PCV13(n = 85)	p-value^a^	PCV7(n = 68)	PCV13(n = 89)	p-value	PCV7(n = 33)	PCV13(n = 100)	p-value
No. Female (%)	14 (44)	40 (47)	0.75	33 (49)	39 (44)	0.56	18 (55)	44 (44)	0.29
Mean age in days at dose (min, max)									
Dose 1	74.9	57.9	0.004	63.8	59.9	0.21	55.8	63.4	0.07
	(42, 211)	(42, 122)		(42, 165)	(30, 127)		(41, 82)	(41, 193)	
Dose 2	149.9	136.1	0.008	133.0	131.6	0.56	132	134.5 (111,	0.52
	(110, 259)	(120, 185)		(107, 248)	(111, 174)		(115, 178)	278)	
Dose 3	217.5	202.8	0.01	198.7	197.2	0.60	199.3	199.3	0.99
	(181, 314)	(182, 259)		(174, 306)	(171, 248)		(181, 237)	(365, 555)	
Dose 4	–	–	–	391.8	377.4	0.002	393.2	379.1	0.02
				(366, 560)	(365, 445)		(366, 574)	(365, 555)	
Mean days between doses (min, max)									
Doses 1 and 2	74.9	78.2	0.40	69.2	71.6	0.41	76.2	71.1	0.12
	(28, 111)	(32, 107)		(29, 104)	(35, 110)		(41, 111)	(41, 111)	
Doses 2 and 3	67.6	66.7	0.82	65.7	65.6	0.98	67.3	64.8	0.42
	(37, 106)	(28, 110)		(29, 106)	(29, 100)		(30, 106)	(28, 100)	
Doses 3 and 4	188.9	188.2	0.95	193.1	180.2	0.006	193.9	179.8	0.008
	(100, 365)	(129, 341)		(137, 379)	(124, 257)		(138, 354)	(140, 257)	
Days between lastdose and blood draw	50.5	39.1	0.003	192.1	179.9	0.009	48.8	28.3	<0.0001
	(15, 94)	(15, 83)		(137, 379)	(124, 257)		(19, 83)	(7, 98)	

a Proportions compared by Fisher’s exact test.

### Comparison of PCV7 and PCV13 Immunogenicity

**Figure 1:**
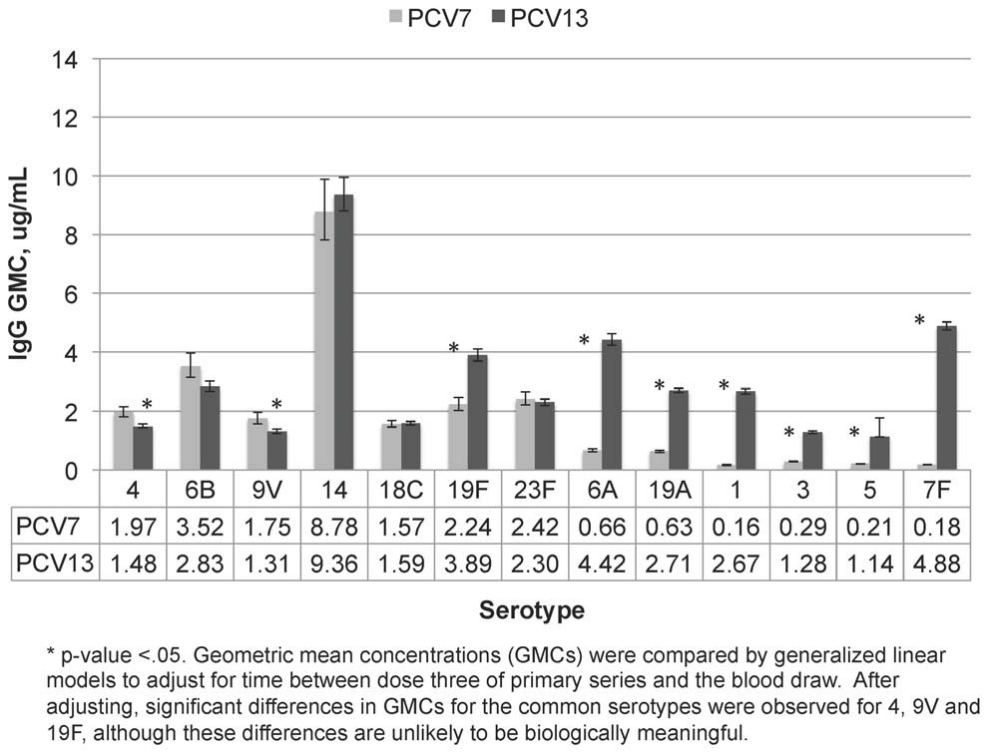
Concentrations of serotype-specific IgG from sera collected post-primary from PCV7 and PCV13 recipients.

**Figure 2:**
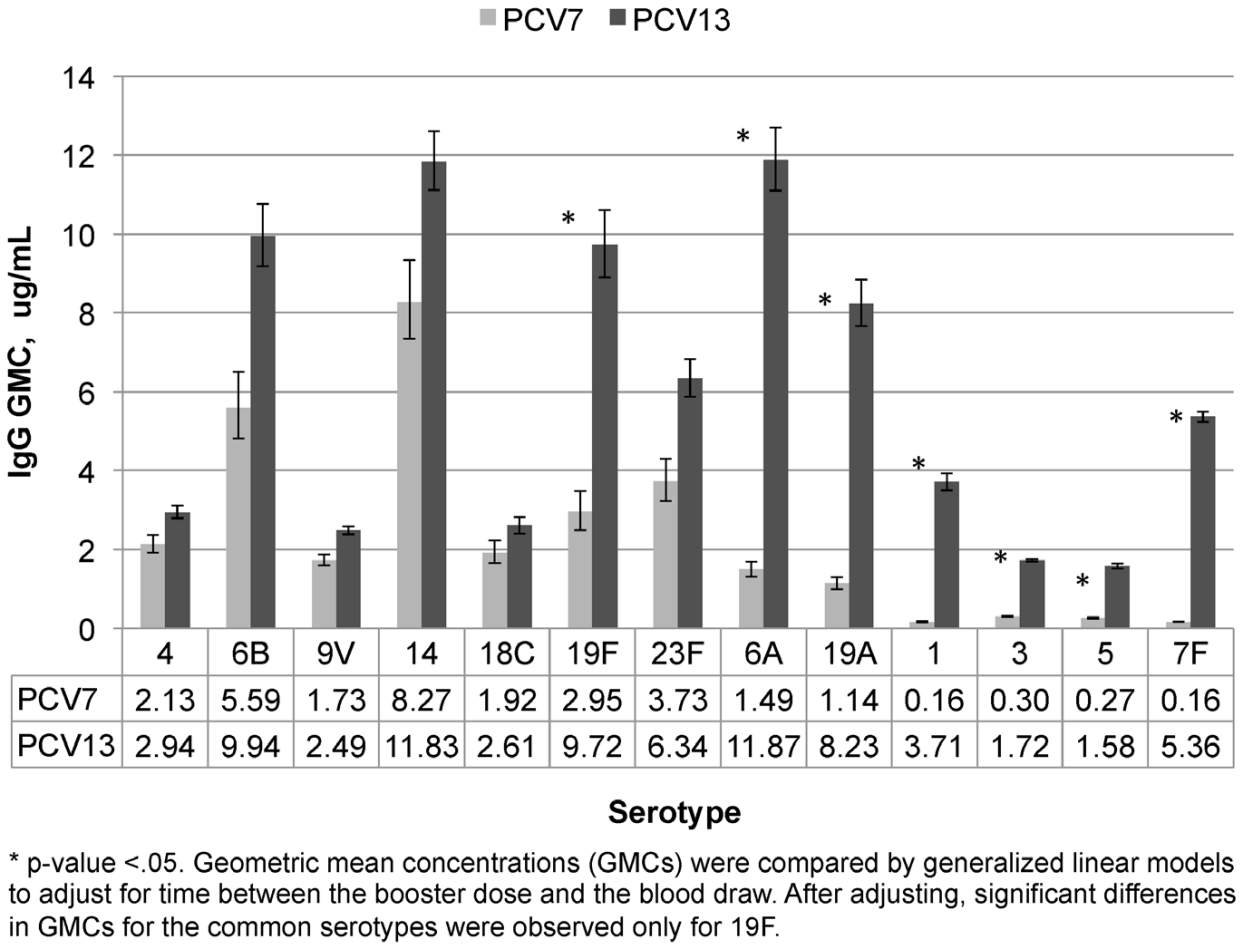
Concentrations of serotype-specific IgG from sera collected post-booster from PCV7 and PCV13 recipients.

Post-primary GMCs for the seven serotypes common between PCV7 and PCV13 (hereafter referred to as “common serotypes”) were significantly lower for serotypes 4 (p = 0.008) and 9V (p = 0.001) and higher for serotype 19F (p = 0.02) among PCV13 participants compared with PCV7 participants after adjusting for differences in the timing of blood collection (Figure 1). Post-primary GMCs for the additional 6 serotypes in PCV13 were significantly higher in the PCV13 group (p≤0.001; Figure 1). Pre-boost antibody levels were lower than post-primary levels for both vaccine groups but all rose sharply following the booster dose irrespective of vaccine type. Among the seven common serotypes, the GMCs following the booster dose were similar between vaccine groups for six serotypes after adjusting for the time between the booster dose and the blood draw (p>0.3); however, post boost GMCs for 19F and the additional 6 serotypes in PCV13 were higher for PCV13 recipients (p≤0.001; Figure 2).

For the common serotypes at least 93% of children who received PCV7 or PCV13 had post-primary and post-boost serotype-specific IgG that reached the threshold used by licensing agencies as a vaccine efficacy correlate (i.e. ≥0.35 *µ*g/mL) (Table 2). When assessing cross reactive serotypes for PCV7 recipients at least 68% achieved IgG ≥0.35 *µ*g/mL for 6A and 19A following primary immunization, which was significantly lower than the PCV13 recipients, but was present in a substantial proportion of PCV7 vaccinated children. Over 78% of PCV7 and 63% of PCV13 recipients had post-primary serotype-specific IgG concentrations ≥1.0 *µ*g/mL for the common serotypes after completing the primary series. A larger percentage of PCV13 children achieved serotype 19F IgG concentrations greater than or equivalent to each of the thresholds after the booster dose compared to PCV7 recipients (p<0.01).

**Table 2:** Percentage of participants who received PCV7 or PCV13 and achieved pneumococcal IgG antibody concentrations ≥0.35 *µ*g/mL or ≥1.0 *µ*g/mL.

		Post primary			Pre-Booster			Post-Booster		
Serotype	Threshold,*µ*g/mL	PCV7%(n = 32)	PCV13%(n = 85)	p-value^a^	PCV7%(n = 68)	PCV13%(n = 89)	p-value	PCV7%(n = 33)	PCV13%(n = 100)	p-value
4	≥0.35	100.0	96.5	0.28	47.1	43.8	0.78	100	99.0	0.56
	≥1	84.4	78.8	0.54	4.4	6.7	0.89	84.8	87.9	0.68
6B	≥0.35	96.9	97.6	0.82	77.9	80.9	0.68	96.9	100	0.08
	≥1	90.6	85.7	0.50	25.0	25.8	0.95	93.8	98.0	0.24
9V	≥0.35	100.0	96.4	0.28	52.9	39.3	0.25	93.8	100	0.01
	≥1	81.3	63.1	0.10	5.9	4.5	0.93	78.1	86.9	0.28
14	≥0.35	100.0	97.6	0.38	97.1	93.3	0.29	100	99.0	0.57
	≥1	96.9	97.6	0.82	76.5	83.1	0.35	100	99.0	0.57
18C	≥0.35	100.0	96.4	0.28	29.4	30.3	0.95	96.9	98.0	0.72
	≥1	78.1	71.4	0.52	0	4.5	–	78.1	83.8	0.51
19F	≥0.35	100.0	100	–	65.7	87.6	0.00	93.8	100	0.01
	≥1	90.6	95.2	0.37	23.9	42.7	0.02	90.6	99.0	0.02
23F	≥0.35	100.0	96.4	0.28	67.6	48.9	0.07	100	99.0	0.57
	≥1	87.5	80.7	0.43	20.6	14.8	0.69	93.8	97.0	0.42
6A	≥0.35	68.8	97.6	<0.0001	45.6	91.0	<0.0001	87.5	100	0.0004
	≥1	34.4	96.4	<0.0001	13.2	52.8	0.03	62.5	99.0	<0.0001
19A	≥0.35	75.0	98.8	<0.0001	70.8	89.8	0.01	82.8	100	<0.0001
	≥1	31.3	90.5	<0.0001	27.7	37.5	0.48	58.6	99.0	<0.0001
1	≥0.35	0	98.8	–	3.3	76.4	0.02	0	100	–
	≥1	0	90.4	–	0	19.1	–	0	91.9	–
3	≥0.35	22.2	96.4	<0.0001	21.8	55.0	0.04	20.0	97.9	<0.0001
	≥1	16.7	63.9	0.10	5.5	12.5	0.73	10.0	73.4	0.02
5	≥0.35	16.1	97.6	<0.0001	14.9	53.9	0.02	34.4	96.0	<0.0001
	≥1	0	59.5	–	0	10.1	–	9.4	73.7	0.43
7F	≥0.35	10.3	100.0	<0.0001	9.1	91.0	<0.0001	3.0	100	–
	≥1	0	98.8	–	0	46.1	–	0	99.0	–

a Proportions compared by Fisher’s exact test.

### Comparison of Functional Antibody Elicited by PCV7 and PCV13

Functional antibody activity (OPA) against 19A and 19F was also assessed in a subset of post-primary sera among PCV7 and PCV13 recipients (Table 3). Serotype 19F OPA titers had a positive correlation with anti-19F IgG ELISA concentrations for both PCV7 (r = 0.67) and PCV13 (r = 0.87). Despite matching for 19F IgG concentration, PCV13 recipients had higher 19F (p = 0.04) opsonic titers compared to PCV7 recipients indicating that 19F antibodies induced by PCV13 are more highly functional than PCV7 induced antibodies. By contrast with serotype 19F, 19A OPA titers and anti-19A IgG were positively correlated (r = 0.49) only among PCV13 recipients. None of the PCV7 recipients demonstrated functional 19A antibody activity (GMT = 8.0).

**Table 3:** Inhibition of serotype-specific 19A or 19F opsonophagocytic activity (GMTs) by purified polysaccharide following PCV7 or PCV13 immunization.

			OPA Results following adsorption with purified polysaccharide							
Serotype	OPA Results Without Adsorption		Serotype 19A polysaccharide adsorption				Serotype 19F polysaccharide adsorption			
	GMT		GMT		% inhibition	p-value^b^	GMT		% inhibition	p-value
	(95% CI)	p-value^a^	(95% CI)	p-value			(95% CI)	p-value		
19A – N = 18 pairs										
PCV7	4.0	0.0002	4.0	0.0002	0	<0.0001	4.0	0.0002	0	0.003
	(4.0–4.0)		(4.0–4.0)				(4.0–4.0)			
PCV13	293.3		25.9		91.1		177.0		39.6	
	(139.9–614.7)		(18.5–36.5)				(88.6–353.6)			
19F – N = 19 pairs										
PCV7	237.9	0.04	168.2	0.05	29.3	0.84	25.9	0.004	89.1	0.61
	(100.8–561.4)		(75.9–372.3)				(17.8–33.2)			
PCV13	547.3		370.8		32.2		34.3		93.7	
	(328.3–912.4)		(215.3–638.8)				(29.9–39.2)			

a GMTs compared by paired t-test or Wilcoxon signed-rank test. In the event that no functional activity was observed, a titer value of 4.0 was assigned as the OPA result. No functional 19A activity was observed for any samples obtained from PCV7 participants resulting in a GMT of 4.0.

b Proportions compared by Fisher’s exact test.

To determine the source of 19F functional antibody activity, sera were adsorbed with 19A polysaccharide and re-tested for 19F functional activity; there were similar reductions in anti-19F activity for PCV7 (29%) and PCV13 (32%; p = 0.84) recipients (Table 3). Having shown that the PCV7 recipients have 19A antibodies but that they are not functional, this finding of equivalent reduction in 19F functional activity was unexpected. When the sera were likewise adsorbed with 19F polysaccharide and re-tested for 19A functional activity, the PCV13 recipients had a 40% reduction in functional activity (the PCV7 recipients had no 19A functional activity even before adsorption) and as expected almost complete inhibition of 19F activity among both the PCV7 and PCV13 recipients.

Turning to serogroup 6, functional serotype 6B responses (OPA titers) were assessed and evaluated for correlation with 6B IgG concentrations; they were highly correlated for PCV13 recipients (r = 0.89) but unexpectedly lacked any linear correlation for sera from PCV7 recipients (r = 0.08). There was no linear correlation between anti-6A IgG concentrations and 6A OPA titers for either PCV7 or PCV13 recipients (r = −0.11 [PCV7]; r = 0.08 [PCV13]). Despite matching on 6B IgG concentrations, PCV7 recipients had higher functional geometric mean 6B antibody titers compared to PCV13 (5558 versus 3221; p = 0.01; Table 4). Serotype 6A and 6C functional antibody titers were higher for PCV13 than PCV7 recipients (6A: 3042 versus 1524, p = 0.01; 6C: 2003 versus 381, p = 0.004; Table 4).

**Table 4:** Inhibition of serotype-specific 6A, 6B and 6C opsonophagocytic activity (GMTs) by purified polysaccharide following immunization with PCV7 or PCV13 vaccine.

Serotype	OPA Resultswithout adsorption		OPA Results following adsorption with purified polysaccharide											
			Serotype 6Apolysaccharide adsorption				Serotype 6Bpolysaccharide adsorption				Serotype 6Cpolysaccharide adsorption			
	GMT(95% CI)	p-value^a^	GMT(95% CI)	p-value	%inhibition	p-value^b^	GMT(95% CI)	p-value	%inhibition	p-value	GMT(95% CI)	p-value	%inhibition	p-value
6A – N = 20 pairs														
PCV7	1523.7(1032.7–2247.8)	0.01	41.4(18.7–91.5)	0.60	97.3	0.74	42.7(19.5–93.1)	0.0002	97.2	0.004	238.5(104.6–543.5)	0.66	84.4	0.59
PCV13	3041.9(1899.9–4870.2)		39.0(28.4–53.4)		98.7		1226.4(644.7–2332.6)		59.7		283.3(159.7–502.3)		90.7	
6B – N = 24 pairs														
PCV7	5558.1(3926.0–7868.6)	0.01	1999.9(1469.8–2721.2)	0.001	64.0	0.47	54.5(29.6–100.6)	0.16	99.0	0.95	2321.9(1750.5–3079.9)	0.005	58.2	0.99
PCV13	3221.4(2122.5–4889.2)		849.5(506.1–1426.0)		73.6		35.6(26.9–46.9)		98.9		1343.0(942.4–1913.8)		58.3	
6C – N = 22 pairs														
PCV7	380.5(133.3–1086.3)	0.004	24.0(10.3–55.7)	0.27	93.7	0.51	27.5(11.7–64.7)	<0.0001	92.8	0.005	22.4(10.2–49.0)	0.27	94.1	0.48
PCV13	3221.4(2122.5–4889.2)		46.6(30.2–71.8)		97.7		895.4(443.4–1807.9)		55.3		36.3(29.9–44.0)		98.2	

a GMTs compared by paired t-test or Wilcoxon signed-rank test.

b Proportions compared by Fisher’s exact test.

The reduction in functional antibody activity following adsorption of sera with 6A, 6B or 6C polysaccharide is shown in Table 4. Homologous polysaccharide adsorption (e.g. 6A adsorption prior to 6A OPA testing) fully eliminated functional activity, as expected and demonstrated the specificity of the antibodies. Adsorption by heterologous polysaccharide had variable effect on OPA activity depending on the vaccine administered and the serotype tested. Notably, serotype 6B adsorption resulted in full reduction in 6A and 6C OPA activity for PCV7 but not PCV13 immunized children. Furthermore, 6A and 6C adsorption resulted in only partial reduction in OPA for 6B among both PCV7 and PCV13 immunized children.

## Discussion

With the replacement of PCV7 by PCV13 in national immunization programs it is critical that data on the likely efficacy of PCV13, particularly in high-risk populations, is rapidly gathered as PCV13 was licensed on immunogenicity alone. This study is the first to assess PCV13 immunogenicity as well as functional antibody activity in a population at high risk for IPD and also the first study to evaluate in detail immunologic cross protection in children when vaccine is administered according to the US national immunization schedule of 2, 4, 6 and 12 months of age.

Although PCV13 recipients were younger at each primary dose than those who received PCV7 (p≤0.01), the IgG response to vaccine was comparable for common serotypes 6B, 14, 18C and 23F and significantly higher for 19F, another serotype common to both products. Greater than 95% of PCV13 recipients met or exceeded the correlate of vaccine efficacy used for licensure (≥0.35 *µ*g/mL) for all serotypes, a proportion similar to that of the older PCV7 recipients for the serotypes common to both products, Furthermore, OPA results of post-primary sera demonstrated that age at receipt of the PCV13 primary series did not affect generation of functional antibody.

The time between the third primary dose or booster dose and the subsequent blood draw was shorter for PCV13 recipients compared to those who received PCV7 (p<0.009). As waning of antibody concentrations would be less between the last dose and blood draw for PCV13 recipients, we adjusted for these differences in the immunogenicity analysis. After adjustment, the GMCs for serotypes 4 and 9V were higher following PCV7 while 19F was higher after PCV13. However, the proportion of recipients with GMCs above the vaccine evaluation threshold was similar for all common serotypes. Therefore, these differences in GMCs are not considered to be biologically relevant.

Following receipt of the booster dose, IgG concentrations to six of the seven common serotypes were similar, which agrees with comparative data previously published for both US and German children [16], [17]. Serotype 19F IgG levels were however superior in our study for the PCV13 recipients at all time points. This might be accounted for by the contribution of cross-reactive 19A antibodies induced by PCV13 but has not been described before. This observation is supported in our study by the higher 19F OPA titers in PCV13 recipients when matched on 19F IgG concentrations and suggests that PCV13 may confer enhanced protection from 19F disease over and above that seen in the PCV7 era. Encouragingly, our results also show that for the seven common serotypes in both vaccines, IgG concentrations were similar compared to previous studies among various populations that have been studied including the same population of American Indian children [18], other children in the United States [19], [20] and other developed [21], [22] and developing countries [23], [24].

The antibody response to the six additional serotypes in PCV13 was superior for PCV13 versus PCV7 at all time points as expected. A significant percentage of PCV7 recipients achieved antibody concentrations for vaccine-associated serotypes 6A and 19A above ≥0.35 *µ*g/mL, presumably through induction of anti-6A and anti-19A IgG by the 6B and 19F conjugate antigens or by natural exposure (for 19A only since there is virtually no 6A colonization in the community). However, the absence of 19A opsonophagocytic activity in PCV7 recipients indicates that these antibodies are not functional against 19A and explains why that disease has continued to rise despite widespread PCV7 immunization in the USA and elsewhere [25]–[30]. Interestingly, adsorption of PCV7 induced sera with purified 19A polysaccharide reduced 19F functional activity, revealing that those same 19A binding antibodies induced by 19F antigen in PCV7 vaccine are functional to some degree against 19F. It is possible that low levels of cross-reacting IgG detected in this way are not sufficient to bring about killing of 19A and higher concentrations, as has been described for 19F, are required [3]. This merits further exploration.

In contrast with 19A, the finding of functional cross-reacting 6A antibodies following PCV7 vaccination is not unexpected as it correlates with clinical observations of near elimination of type 6A disease and colonization following widespread PCV7 use [31]. Detection of functional serotype 6C activity in the PCV7 recipients is surprising given that there has been little impact on 6C colonization or disease following PCV7 use [4], [32], [11] and that immunochemically 6B induced antibodies are not predicted to bind 6C [33]. This 6C cross-reacting phenomenon following PCV7 or PCV13 has been described before [31], [34]. However, in our study 6C functional titers following PCV7 were significantly lower than those seen following PCV13. Following PCV13, both 6B and 6A induced antibodies combine to cross react with 6C, which is reflected in the fact that 6B adsorption completely inhibited PCV7 sera from killing 6C but only reduced killing by 50% in the PCV13 recipients. It is conceivable that these higher 6C functional titers following PCV13 could have an impact on 6C disease. This is further suggested by colonization studies that showed reductions in 6C carriage among PCV13 recipients including in this American Indian population [35], [36]. More complete observations and formal analysis from colonization and disease surveillance will be coming from a variety of countries in the near future.

Sera from PCV7 and PCV13 post-primary recipients were matched on 6B IgG concentrations to minimize the impact of differences in 6B IgG concentration on comparisons of functional antibody activity. In spite of this matching, the functional activity of serotype 6B antibody was higher for PCV7 recipients compared to children who received PCV13. Thus, gram-for-gram, PCV7-induced 6B antibody appear to be more functional than PCV13 induced antibody; similar results were observed by Cooper et al. [34] comparing PCV7 and PCV13 6B OPA titers. The reason for this difference is unclear as PCV13 contains slightly more purified 6B polysaccharide (4.4 µg per dose compared to 4.0 µg in a dose of PCV7), slightly more carrier protein CRM_197_ (34 µg compared to 20 µg per dose of PCV7) and identical amounts of aluminium phosphate (125 µg as aluminium phosphate). Changes in PCV7 and PCV13 manufacturing including conjugation or interference from the additional serotypes may account for the difference.

Pneumococcal conjugate vaccines have had a significant impact on reducing IPD and colonization caused by vaccine serotypes in populations at high risk of IPD such as American Indians. Functional analyses of the vaccine serotype and cross-reactive antibodies demonstrate that their function is less specific than originally understood. Our analyses indicate that PCV13 could provide enhanced protection against serotypes included in the vaccine (i.e. 19F) and those not present in the vaccine (i.e. 6C) compared to that seen with PCV7 although for other serotypes (i.e. 6B) equivalence with PCV7 will need to be monitored. Comparisons of IPD and pneumococcal colonization trends before and after conjugate vaccine introduction can contribute to our understanding of the population-level impacts of PCV on both immunized and un-immunized persons. Because there are a significant number of pneumococcal serotypes that are clinically relevant, functional analyses such as this one are important tools to improve understanding the contribution of PCV products to disease reduction. Furthermore, studies such as this help to focus attention on potential risks associated with product switches, and provide the biologic basis for these clinical disease reduction observations.

## Supporting Information

Figure S1 **Summary of sera included in analyses.**
(TIFF)
