# Supplementary figures and images for: Comparative Immunogenicity of 7 and 13-Valent Pneumococcal Conjugate Vaccines and the Development of Functional Antibodies to Cross-Reactive Serotypes
Source: PLoS One. 2013 Sep 23;8(9):e74906. doi: 10.1371/journal.pone.0074906 (PMC3781100; doi:10.1371/journal.pone.0074906)

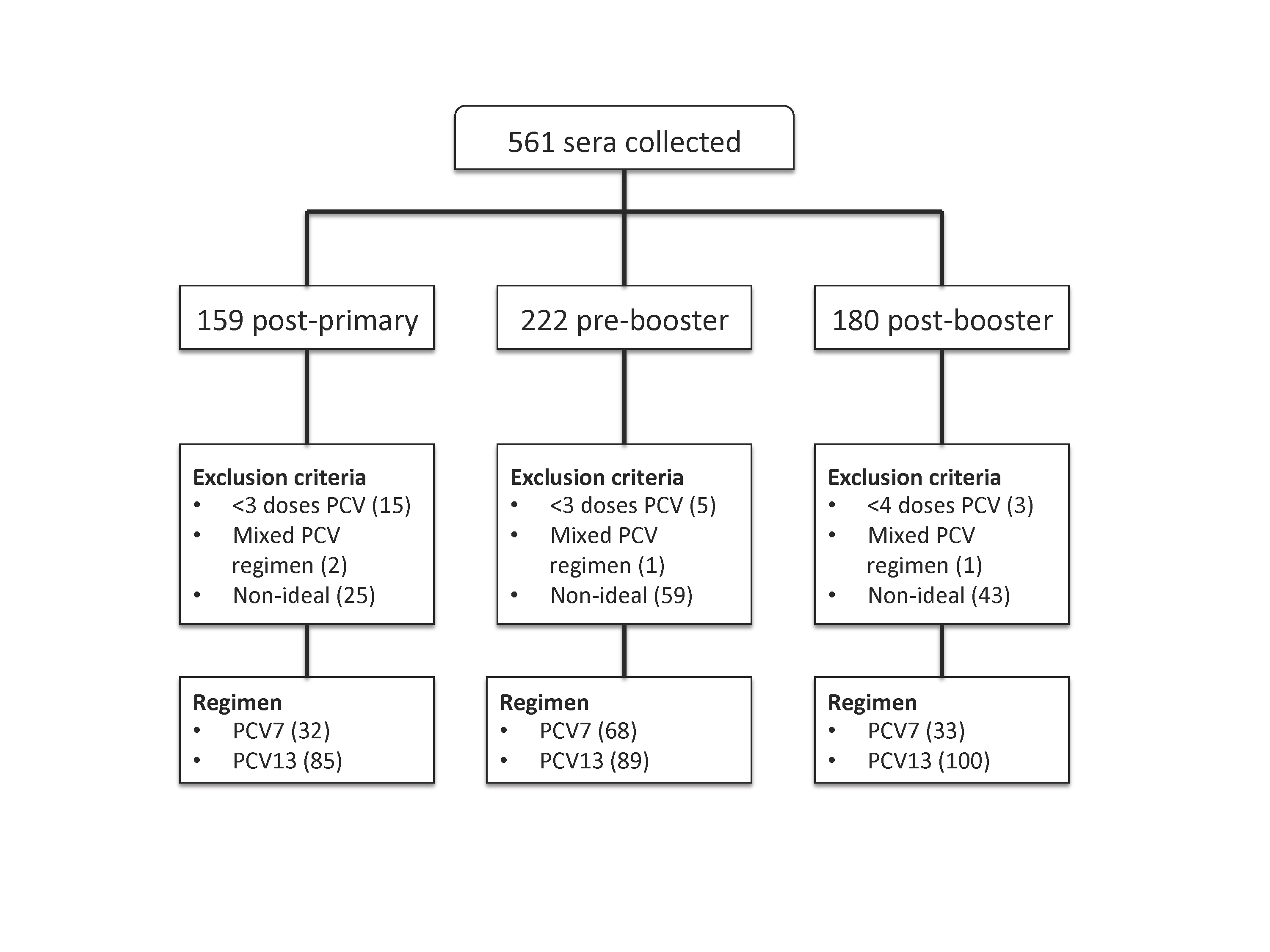

Supplement: Figure S1 — Summary of sera included in analyses. (TIFF) [file pone.0074906.s001.tiff]
